# A Cross-Sectional and Longitudinal Analysis of Pre-Diagnostic Blood Plasma Biomarkers for Early Detection of Pancreatic Cancer

**DOI:** 10.3390/ijms232112969

**Published:** 2022-10-26

**Authors:** James Mason, Erik Lundberg, Pär Jonsson, Hanna Nyström, Oskar Franklin, Christina Lundin, Peter Naredi, Henrik Antti, Malin Sund, Daniel Öhlund

**Affiliations:** 1 Department of Radiation Sciences, Umea University, 901 87 Umea, Sweden; 2 Wallenberg Centre for Molecular Medicine, Umea University, 901 87 Umea, Sweden; 3 Department of Surgical and Perioperative Sciences, Umea University, 901 87 Umea, Sweden; 4 Department of Chemistry, Umea University, 901 87 Umea, Sweden; 5 Department of Surgery, Institute of Clinical Sciences, University of Gothenburg, 413 45 Gothenburg, Sweden; 6 Department of Surgery, University of Helsinki and Helsinki University Hospital, 000 29 Helsinki, Finland

**Keywords:** carcinoma, pancreatic ductal, biomarkers, tumor, early detection of cancer, tumor microenvironment, analysis

## Abstract

Pancreatic ductal adenocarcinoma (PDAC) is a major cause of cancer death that typically presents at an advanced stage. No reliable markers for early detection presently exist. The prominent tumor stroma represents a source of circulating biomarkers for use together with cancer cell-derived biomarkers for earlier PDAC diagnosis. CA19-9 and CEA (cancer cell-derived biomarkers), together with endostatin and collagen IV (stroma-derived) were examined alone, or together, by multivariable modelling, using pre-diagnostic plasma samples (*n* = 259 samples) from the Northern Sweden Health and Disease Study biobank. Serial samples were available for a subgroup of future patients. Marker efficacy for future PDAC case prediction (*n* = 154 future cases) was examined by both cross-sectional (ROC analysis) and longitudinal analyses. CA19-9 performed well at, and within, six months to diagnosis and multivariable modelling was not superior to CA19-9 alone in cross-sectional analysis. Within six months to diagnosis, CA19-9 (AUC = 0.92) outperformed the multivariable model (AUC = 0.81) at a cross-sectional level. At diagnosis, CA19-9 (AUC = 0.995) and the model (AUC = 0.977) performed similarly. Longitudinal analysis revealed increases in CA19-9 up to two years to diagnosis which indicates a window of opportunity for early detection of PDAC.

## 1. Introduction

The five-year relative survival rate of pancreatic ductal adenocarcinoma (PDAC) is merely 11% [1], and it is projected to become the second deadliest cancer by 2030 [2]. Radical surgical resection remains the only cure for PDAC, but only a minority of patients have resectable disease at diagnosis. In general, systemic treatments only add small survival benefits, although new chemo-intensive regimens have been introduced [3,4]. There are no good treatment-predictive markers and, compared to other solid cancers, very few targeted treatment options have emerged. Early diagnosis is imperative, as indicated by the large discrepancy between the relative five-year survival rates of localized (42%) and metastasized disease (3%) [1]. Early PDAC is associated with unspecific symptoms and, by the time they develop, metastasis has often already occurred [1,5].

A previous study indicated a six–eight-year time frame between localized and metastatic PDAC by mathematical modelling of PDAC sub-clone genomes [6]. More recently, it was demonstrated by analyzing deoxyribonucleic acid (DNA) copy numbers and gene rearrangements that PDAC may undergo more rapid progression [7], which was supported by another study estimating PDAC progression based on age at different stages [8]. Consequentially, the window of opportunity for early detection might be narrower than expected and indicates that treatment at the time of clinical symptoms is likely futile for the majority. Taken together, the model of PDAC progression suggests that it is possible to develop a method for identifying PDAC at a potentially curable stage, if efficient tumor markers are available.

The only clinically applicable circulating PDAC biomarker is the glycosylation epitope carbohydrate antigen 19-9 (CA19-9), mainly used during patient follow-up, due to insufficient sensitivity and specificity as a diagnostic marker [9]. By combining CA19-9 with other markers, including carcinoembryonic antigen (CEA), diagnostic accuracy has been improved [10]. This suggests that a panel of several markers with sufficient diagnostic accuracy might result in an effective population screening method. Many potential PDAC biomarkers currently have problems with lack of validation in independent cohorts, insufficient sensitivity and specificity, or difficulty in discriminating between malignant and benign pancreatic disease [11].

One difficulty in tumor marker development and validation is cellular heterogeneity within and between tumors [6]. Abundant stroma is characteristic of PDAC and constitutes the vast majority of PDAC masses [12]. Turnover of stromal substances, such as endostatin and collagen IV, can be detected in blood and reflect disease stage, which has led to the hypothesis that these molecules may be valuable biomarkers [13,14,15].

If there exists a window for early detection, then there may be an opportunity to use effective diagnostic markers in a pre-diagnostic setting to detect PDAC earlier. Furthermore, sensitivity and specificity could be improved by modelling several different markers together, where a combinatorial expression pattern may be more powerful than any marker in isolation. Here, we explore the use of cancer cell biomarkers (CA19-9 and CEA) together with stroma-associated PDAC markers (collagen IV and endostatin), both individually and together for early PDAC detection. We examined pre-diagnostic plasma samples collected from the Northern Sweden Health and Disease Study (NSHDS) [16] biobank, which contains samples collected during a population-based health intervention from individuals at 40, 50, and 60 years of age, including those of future PDAC patients. Cross-sectional analyses of the NHSDS samples are compared to analysis of samples collected at diagnosis from the Umeå Prospective Clinical Biobanks (UPCB). Finally, we used the NHSDS samples to explore longitudinal changes in biomarker expression to identify meaningful trends within individual future PDAC patients that may not be clear at a cross-sectional population level.

## 2. Results

### 2.1. NSHDS Cohort Description

**Figure 1:**
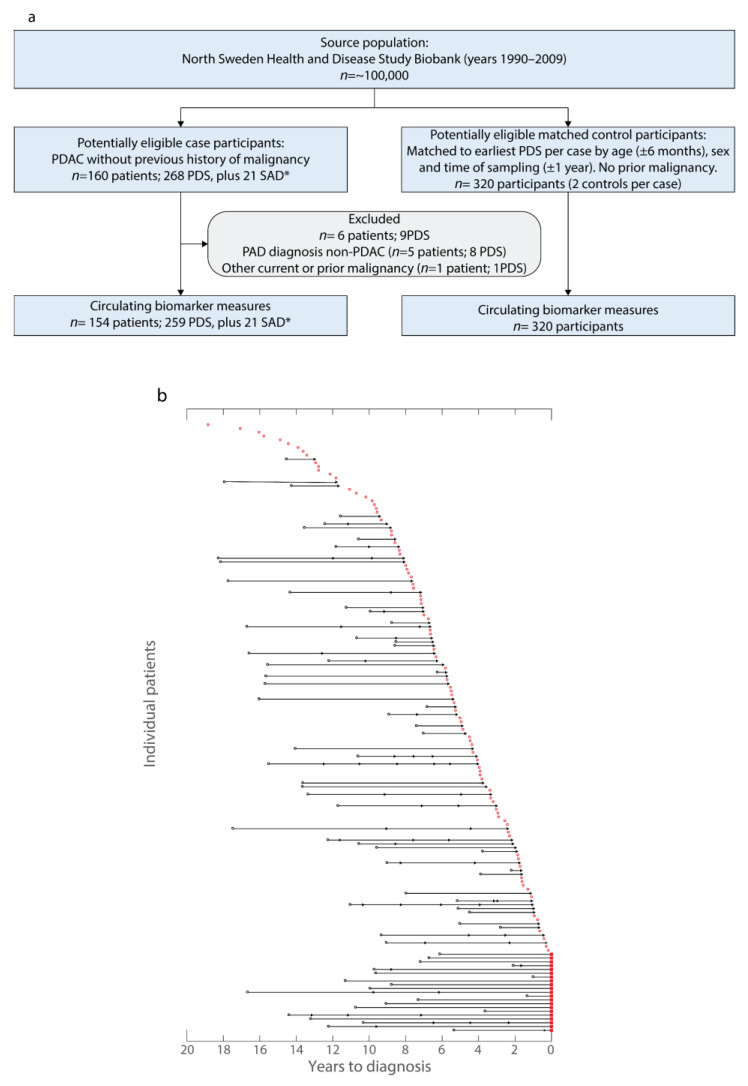
Description of the pre-diagnostic cohort from Northern Sweden Health and Disease Study (NSDHS) and of those patients that also provided samples at diagnosis (SAD) in the Umeå prospective clinical biobanks (UPCB). (**a**) Flowchart of the study and matched control cohorts from the NSHDS cohort showing the inclusion and exclusion criteria (PDS: Pre-diagnostic sample; SAD: Sample at diagnosis collected from the UPCB; * samples collected within 40 days from diagnosis date prior to any surgical or oncological treatment). (**b**) Waterfall plot illustrating relationship between included samples and time of clinical diagnosis. Each future PDAC patient is represented on the y-axis. All unconnected dots represent samples from future PDAC patients that provided only one sample. All connected points are from future PDAC patients that provided multiple samples. The shape of each dot indicates the respective order of samples from each patient (circle, earliest sample; arrowhead, subsequent pre-diagnostic sample(s); square, sample at PDAC diagnosis). Samples in black were those used in both OPLS-DA modelling in the longitudinal analyses and cross-sectional analysis. Samples in red were used for cross-sectional analysis. Patients that provided samples at diagnosis (squares) had all provided samples examined by spaghetti plots.

Overall, 154 future PDAC patients (cases) fulfilled the inclusion but not exclusion criteria and were matched to two controls per case (Table 1, Figure 1a). Cases and controls did not demonstrate any significant differences for age, sex, or smoking status (Table 1). Pre-diagnostic samples were collected from cases ranging from 18 years to within one year prior to PDAC diagnosis (Table 1, Figure 1b). Of the 154 cases, 99 contributed one pre-diagnostic sample and the remaining 61 cases contributed multiple pre-diagnostic samples (Figure 1b). In total, 259 pre-diagnostic samples (NSHDS), 320 control samples (NSHDS), and 21 samples at diagnosis (UPCB, Figure 1b) were available to test the predictive and diagnostic biomarker capacities.

### 2.2. Cross-Sectional Analysis

**Figure 2:**
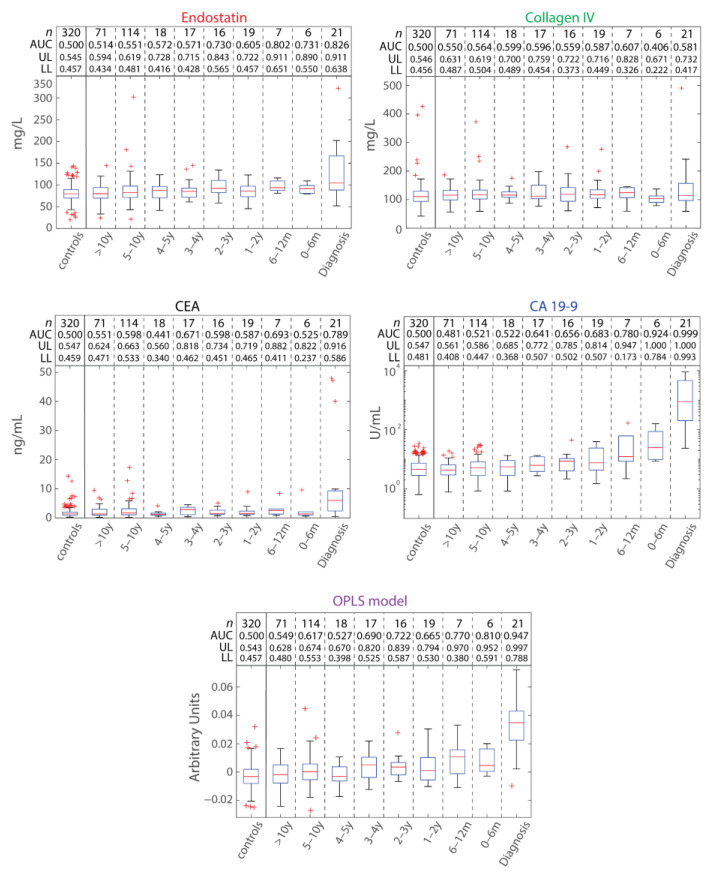
Separating future PDAC cases from controls in pre-diagnostic and diagnostic samples with a cross-sectional analysis. Box plots of the individual marker concentrations and OPLS-DA model scores (y-axes) for controls and (future) PDAC patients, indicating the median (red line), upper and lower quartiles (box), inter-quartile range (whiskers), and outliers (red cross). Controls and samples at diagnosis are categorized on the x-axis and pre-diagnostic samples are stratified in the x-axis according to time prior to diagnosis (0–6 months, 6–12 months, 1–2 years, 2–3 years, 3–4 years, 4–5 years, 5–10 years, and >10 years). The number of samples (*n*), area under the curve (AUC), as well as the 95% confidence interval (UL, upper limit; LL, Lower limit) values for each x-axis category are indicated.

Circulating plasma levels of CA19-9, CEA, collagen IV, and endostatin were evaluated to identify whether any individual marker could predict future PDAC cases by receiver operating characteristic (ROC) analysis (Figure 2). Cases were stratified according to time to diagnosis. No individual marker performed as well as CA19-9 at, or before, diagnosis. CA19-9 demonstrated an area under the ROC curve (AUC) above 0.9 within six months to diagnosis, and 0.999 for those 21 samples provided at diagnosis. Endostatin demonstrated an AUC of 0.731 within six months to diagnosis and an AUC of 0.826 at diagnosis. Neither CEA nor collagen IV demonstrated any discrimination between future patients and controls within six months to diagnosis. At diagnosis, CEA had an AUC of 0.789, whilst collagen IV demonstrated little discriminatory power (AUC = 0.581).

**Figure 3:**
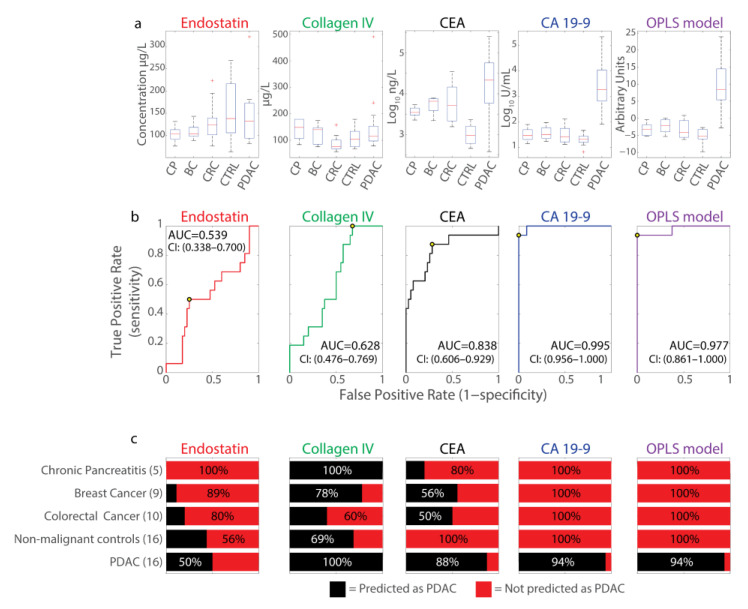
Separating PDAC cases from controls with other cancer forms, inflammatory disease, and non-malignant diseases at the time of diagnosis in the Umeå prospective clinical biobanks (UPCB) cohort. (**a**) Box plots of the measured EDTA plasma biomarker values from samples provided by patients or controls. Median values are indicated by the red line in the box, and values outside plotted whiskers are indicated by a red cross. Y-axis plotted linearly except for measures from CEA and CA19-9, which are on a log_10_ scale. CP, chronic pancreatitis; BC, breast cancer; CRC, colorectal cancer; CTRL, non-malignant controls; PDAC, pancreatic adenocarcinoma. (**b**) ROC curves displaying sensitivity and false positive rate (1-specificity) of measured circulating biomarker levels (endostatin, collagen IV, CEA, and CA19-9) and a multivariate OPLS-DA model combining all four markers for PDAC detection. Samples were measured from PDAC patients at time of diagnosis (*n* = 16) and compared against other controls (*n* = 40, Table 2) for the analysis, including patients with breast cancer (*n* = 9), colorectal cancer (*n* = 10), CP (*n* = 5), or with non-malignant disease (*n* = 16). Area under the curve (AUC) is specified in each graph and the optimal cut-off point indicated with a circle. (**c**) Percentage of individuals predicted to have PDAC (in black) or not (in red) for the different markers and the OPLS-DA model for each patient category using the optimal cut-off.

A similar approach using only samples collected from patients at diagnosis in the UPCB (Table 2) demonstrated similar findings. Here, both CA19-9 and CEA demonstrated marked increases in plasma concentration compared to healthy controls (Figure 3a). AUCs for both markers were above 0.8 (Figure 3b); however, only CA19-9 elevation was seen specific to PDAC without much elevation in the other cancers (breast cancer and colorectal cancer) or chronic pancreatitis (CP) examined (Figure 3a–c). Consequently, both CEA (Sensitivity = 0.875, specificity = 0.725) and CA19-9 (sensitivity = 0.938, specificity = 1.00) could identify 100% of healthy controls as being without PDAC, but only CA19-9 was specific enough not to mischaracterize patients with other diagnoses as having PDAC (Figure 3c). Endostatin had an AUC of 0.539 at diagnosis and poor PDAC predictive capacity (sensitivity = 0.500, specificity = 0.750) (Figure 3b,c). As with the NSHDS cohort, collagen IV demonstrated little discriminatory power in UPCB samples (AUC = 0.628), although being sensitive to, but with very little specificity for, PDAC (sensitivity = 1.00, specificity = 0.325) (Figure 3b,c).

Taken together, CA19-9 outperformed other markers for PDAC sensitivity and specificity from samples at diagnosis. CEA and endostatin each demonstrated some discriminatory power at diagnosis; however, their efficacy was cohort-dependent. Finally, only CA19-9 could discriminate future patients from controls, but only within six months to diagnosis.

Whether some combinatorial pattern of marker expression may predict future PDAC cases was explored by multivariable modelling. An orthogonal projections to latent structures discriminant analysis (OPLS-DA) model was constructed and examined by cross-sectional analysis in the same way as the individual markers. The OPLS-DA model outperformed endostatin, collagen IV, and CEA, but it did not perform better than CA19-9 at any time (Figure 2). Similarly, when the same OPLS-DA modelling approach was applied to the samples unique to the UPCB, the OPLS-DA model performed similarly to CA19-9 alone (Figure 3a–c).

Overall, cross-sectional analysis of the markers individually or in combination could not predict future PDAC cases by cross-sectional analysis, except for CA19-9 at diagnosis and within six months to diagnosis.

### 2.3. Longitudinal Analysis

**Figure 4:**
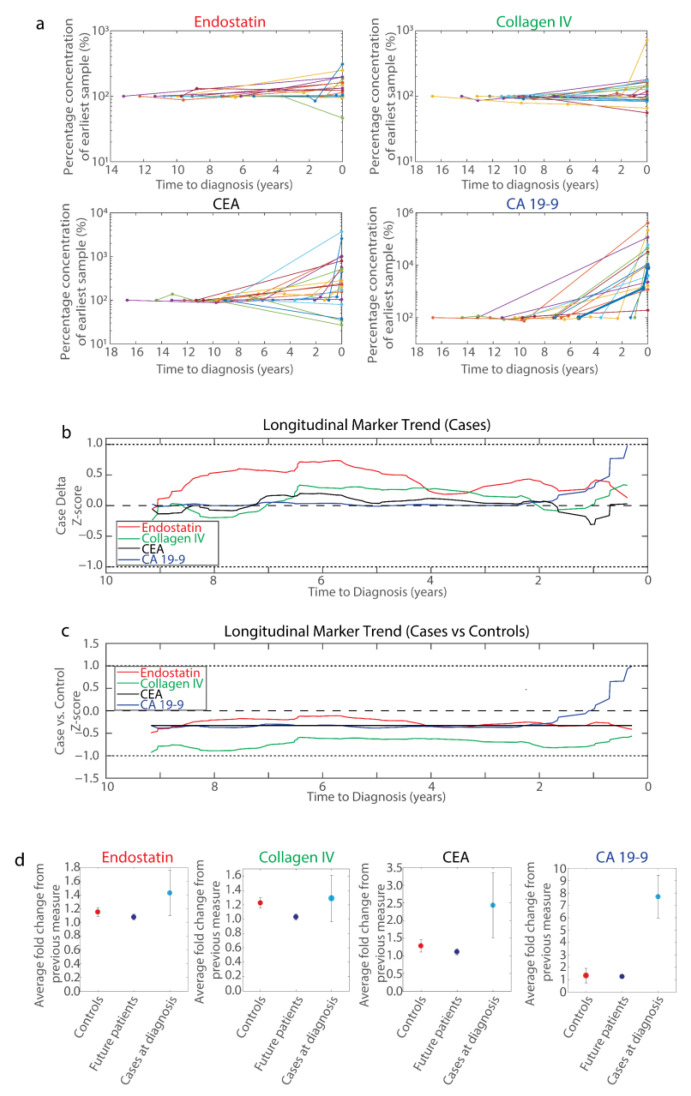
Longitudinal examination of blood plasma biomarker fluctuations. (**a**) Spaghetti plots of blood plasma biomarker concentrations (top left, Endostatin (*n* = 18); top right, Collagen IV (*n* = 20); bottom left, CA19-9 (*n* = 16); bottom right, CEA (*n* = 20)) normalized to the first pre-diagnostic sample per patient (%) against time to diagnosis (years) for patients that provided both samples at diagnosis and pre-diagnostic samples (that is, those patients that provided samples at diagnosis in Figure 1b). Different colored lines represent different patients. In the CA19-9 panel, one patient demonstrated elevated circulating CA19-9 levels prior to diagnosis, but no elevated levels for the other markers (blue, bold). (**b**) Longitudinal changes in blood plasma biomarkers in future PDAC patients with respect to marker fluctuations of successive samples. Plotted for each marker are the mean change in marker expression for 89 chronologically successive batches of 20 future patient delta samples divided by the standard deviation of all future patient delta samples (Case delta Z-scores, y-axis) against the average time to diagnosis of the latest delta sample in each batch of 20 deltas (x-axis). Each line was generated by applying a Savitzky–Golay filter to these data points. Values greater than zero indicate that there was an increase in marker expression from the previous measure in future patients, and the converse for values less than zero. (**c**) Longitudinal changes in blood plasma biomarkers of future PDAC patients in context of marker fluctuations in healthy subjects. Points were plotted similarly as in (**b**), except that the mean delta for all control subjects was subtracted from each batch of 20 patient deltas and then divided by the standard deviation of all control deltas (Case vs. Control Z-score, y-axis). All longitudinal changes in control subjects were compared to each set of 20 deltas described for the cases, since there is no time to diagnosis for control subjects. Values greater than zero indicate an increase in marker expression from the previous measure in future patients relative to the change in marker expression of control samples, and the converse for values less than zero. (**d**) A comparison of fold changes in blood plasma biomarkers between sampling times per subject in controls (red), future cases prior to diagnosis (blue), and change in levels for cases at diagnosis from their most recent measure prior to diagnosis (cyan). Dot plot indicates the mean fold change in sampling from last sample, with the error bars representing a 95% confidence interval.

Of the 154 future PDAC patients identified from the NSHDS biobank, 21 contributed samples at diagnosis to the UPCB (Figure 1b). Thus, the relative change in marker expression from these cases could be measured over time up to and including diagnosis (Figure 4a). Here, pronounced elevation in marker expression occurred very late and was generally seen at diagnosis, if at all. In one case, elevated CA19-9 levels were observed prior to diagnosis; however, this was within six months to their diagnosis (bold blue line, Figure 4a). That said, variation in marker expression prior to diagnosis could be observed, indicating that marker fluctuations within individuals over time may be indicative of future PDAC diagnosis.

Altogether, 61 cases contributed multiple pre-diagnostic samples, which permitted a longitudinal examination of marker fluctuation over time (Figure 4b). For these cases, the Z-scores for successive differences in marker expression over time with respect to diagnosis were calculated to identify trends in marker expression changes compared to baseline within individual cases. Endostatin appeared to consistently increase between measurements in future cases, whereas all other markers demonstrated a lower increase; however, the mean delta never exceeded one standard deviation for any marker. CA19-9 values did not fluctuate until around two years prior to diagnosis, when CA19-9 expression began elevating, exceeding the increase seen in any other marker by one year prior to diagnosis. From around six months prior to diagnosis there were insufficient data points for analysis.

To account for marker fluctuation that may occur canonically throughout life, a similar analysis that accounted for change of marker expression over time within healthy controls was performed (Figure 4c). Here, the general increase in endostatin identified in future patients also occurred within healthy controls, thus flattening the baseline fluctuation in endostatin (Figure 4c). This was similar for all examined markers, indicating little shift from baseline over time. However, CA19-9 retained a steep increase in mean marker expression from around two years prior to diagnosis, which was a shift from the stable baseline of the preceding years.

Curiously, the mean delta for all markers when incorporating control measures was less than zero, except for CA19-9 from one year to diagnosis (Figure 4c). Further examination identified that all subjects, both controls and future patients, exhibited a general increase in circulating biomarker expression when compared to their previous measure; however, for all biomarkers, this increase appeared greater in the controls than in future patients (Figure 4d). This trend was inverted at the time of diagnosis, where there was a substantial increase in all markers in patients compared to their most recent pre-diagnostic level.

Overall, there was little evidence that longitudinal changes in endostatin, collagen IV, and CEA hold predictive value in advance of PDAC diagnosis. In contrast, CA19-9 demonstrated increasing longitudinal marker expression from around two years to diagnosis (Figure 4b,c). In general, these biomarker levels increase, if at all, very close to PDAC diagnosis, as illustrated by the changes in biomarker levels from patients with both pre-diagnostic samples and samples at diagnosis (Figure 4a). Of these cases, only one case had elevated CA19-9 prior to diagnosis (<6 months, Figure 4a), indicating that cases where CA19-9 increases within two years to diagnosis may be uncommon, but the increase large enough to affect grouped data, suggesting a subset of cases that could be identified years earlier.

## 3. Discussion

Diagnosis at an earlier stage might improve PDAC survival, with more patients undergoing radical surgical resection. Here, we examined four promising or clinically utilized biomarkers individually and combined in a pre-diagnostic setting using samples collected prior to PDAC diagnosis. In samples acquired at or very close to diagnosis, accurate differentiation was possible, but when analyzing samples collected beyond six months to diagnosis, no biomarker nor a combined model could predict future disease in a cross-sectional setting. However, there is evidence by longitudinal analysis that CA19-9 begins to increase in some individuals from around two years prior to diagnosis.

The markers examined included cancer cell-derived CEA and CA19-9, as well as the stromal substances collagen IV and endostatin, which have previously been suggested as promising PDAC biomarkers [13,14,15,17]. By combining stroma-derived and cancer cell-derived biomarkers, we tested the hypothesis that a combination of markers that reflect different tumor aspects improves sensitivity and specificity compared to individual markers, which in turn could be applicable for pre-diagnostic testing. Therefore, these markers were tested both separately and in combination by multivariable modelling in a pre-diagnostic setting examining NSHDS samples of future PDAC cases compared to matched controls (Figure 2). Of the individual markers, none demonstrated AUCs that were above 0.9 apart from CA19-9 from within six months to diagnosis. The combinatorial model of the markers did not determine a pattern in marker expression that improved AUCs compared to CA19-9 alone, neither pre-diagnostically nor with samples available at diagnosis. Consequently, these data suggest the earliest available window for discriminating future PDAC cases from controls in a cross-sectional setting to be within six months to diagnosis by CA19-9 expression. This narrow window is suboptimal for a population screening regimen and would likely not allow for diagnosis at a stage that would change the clinical outcome.

That the multivariable model performed comparably to, but not better than, CA19-9 alone was verified with an independent cohort (Figure 3). Here, specificity for PDAC was also tested with respect to other cancer types and CP, since discriminating from healthy controls is insufficient for PDAC specificity. For example, glypican-1 in circulating exosomes demonstrated discrimination between PDAC patients and healthy controls with 100% sensitivity and specificity; however, breast cancer patients also display high values indicating insufficient PDAC specificity [18]. Overall, at diagnosis, CA19-9 was the most sensitive and specific marker, consistent with it being the current gold-standard marker for PDAC [19]. CEA, meanwhile, had lower sensitivity than CA19-9 at detecting PDAC, but similar or slightly greater specificity [19]. CEA was accurate in predicting healthy controls but struggled to distinguish PDAC from CP and other cancer types (Figure 3c), likely due to the association of CEA with these maladies [20,21,22]. Collagen IV demonstrated high sensitivity for PDAC, but low specificity, and endostatin was least sensitive for PDAC diagnosis in the UPCB cohort. This contrast for endostatin appears related to the greater variance in measures from non-PDAC UPCB controls compared to measures from NSHDS controls (Figure 2 and Figure 3a). Finally, the combinatorial model of all four individual markers performed comparably to CA19-9 and discriminated PDAC patients at diagnosis from healthy and unhealthy controls, although its performance was likely driven by CA19-9 (Figure 3c).

The cross-sectional analysis examined marker expression with respect to matched controls at a snapshot in time and consequently did not account for patterns in marker fluctuations within individuals over time, which may be informative. Therefore, a longitudinal examination of mean marker expression fluctuations over time was performed (Figure 4b), which indicated that future patients have a slight increase in endostatin levels over time, but that this increase is canonical with ageing, since it is nullified by comparing to endostatin fluctuations in healthy controls (Figure 4c). Furthermore, all marker trends over time in future patients were less varied when compared with variance in healthy controls, except for CA19-9 from around two years prior to diagnosis when mean CA19-9 levels markedly increased. This increase was small and within one standard deviation of that seen in controls, and, consequentially, likely insufficient for clinical application.

The finding of an increase in CA19-9 from around two years to diagnosis is also reflected in plasma levels of CA19-9 by cross-sectional analysis, where the upper quartile begins to increase relative to previous measurements (Figure 2). The median value remains unchanged, indicating that the increase is a result of increased levels of individual cases, which in turn suggests that there is a subset of future cases that demonstrate elevated levels of CA19-9 prior to diagnosis. This observation is supported by the fact that those 21 cases for whom there were data at diagnosis, only one demonstrated an elevated CA19-9 value compared to past samples (Figure 4a).

A two-year timeframe is similar to another finding where CA19-9 was elevated in 16% of future PDAC patients up to three years prior to diagnosis and that most CA19-9 ‘change-points’ occurred within 12 months to diagnosis [23], a finding comparable to ours (Figure 2 and Figure 4a,b). An additional study identified that detection of future PDAC patients could be moderately improved up to 18 months prior to PDAC diagnosis by using CA19-9 in conjunction with apolipoprotein A2 [24]. Still, sensitivity remained low prior to diagnosis [23] and the positive predictive value for CA19-9 was only 0.5–0.9% in previous prospective studies on asymptomatic populations [25]. Regardless, CA19-9 is elevated relatively late in PDAC progression.

Future studies may discover novel biomarkers or combinations that can accurately predict PDAC early. However, this possibility assumes a slow progression from dysplasia to metastatic disease. Although the etiology of PDAC remains unsettled [6,7], currently, no biomarkers have been identified with any predictive capacity greater than a few years. Identification of biomarkers with predictive capacity much earlier may exist, but they may not be those that perform well at diagnosis. Numerous studies have presented potential PDAC biomarkers based on good discrimination at diagnosis [26]. However, having markers with good discriminatory capacity at the time of diagnosis, even in stage 1–2 patients, does not necessarily translate to the pre-diagnostic setting. Franklin et al. recently found a panel of 15 miRNAs with superior diagnostic capacity to CA19-9 at PDAC diagnosis, but when tested in a pre-diagnostic cohort of future PDAC patients, the AUC dropped to 0∙60–0∙65 [27]. This highlights the importance of evaluating PDAC biomarkers using pre-diagnostic samples and future PDAC biomarker studies should aim at conducting unbiased multi-omics analysis in a pre-diagnostic setting with subsequent validation.

It is also worth considering that a longitudinal population screening program would confer unique challenges compared to single-instance screening assays, including both logistical and fiscal. These challenges could be offset by limiting screening programs to high-risk populations, such as those affected by familial pancreatic cancer, for whom infrastructure for regular screening already exists [28]. Current surveillance assays for high-risk individuals, such as invasive endoscopic ultrasonography and magnetic resonance imaging, are also comparatively costly compared to ELISA-based assays. Consequently, an effective ELISA panel could represent a reduction in financial investment. Regardless, the capacity to identify future pancreatic patients, even if by six months, represents an approximate doubling in median life expectancy from diagnosis for the patient [1,29]. Whilst the etiology of PDAC remains unclear, an additional six months could also be the difference between catching PDAC at a resectable stage or not, which presently constitutes the only effective treatment.

Taken together, our study and others indicate that clear increases in biomarker expression occur late in disease progression, close to the time of diagnosis [23,24]. The most sensitive and specific marker was CA19-9, which at a cross-sectional level increased considerably within just 6 months to diagnosis. The construction of a model including other biomarkers, both stromal- and cancer cell-derived, did not perform better. However, there is accumulating evidence that there are subtle increases in PDAC biomarker levels up to several years prior to diagnosis when examined longitudinally within an individual [23,24]. The sudden change in marker expression that occurs around the time of diagnosis could also be a clue as to the etiology of PDAC, supporting the idea that, whether or not initial cancer clones appear early, there is an exponential development phase just months before clinical signs appear, at which point changes in circulating biomarkers can be detected. Using elevated circulating proteins as early detection biomarkers for PDAC appears difficult, although holistic approaches yield some promise [30], where recently a panel of 29 serum biomarkers was identified that could distinguish stage I and II PDAC patients from controls using samples at diagnosis. In addition, metabolomic approaches provide another approach with high resolution that may uncover metabolite panels for PDAC diagnosis that in future could be developed into a pre-diagnostic setting [31]. In contrast, if clones do develop early, then there is additional opportunity for early PDAC detection by means of low-level, amplifiable material such as circulating tumor DNA [32].

## 4. Materials and Methods

### 4.1. Ethical Considerations

The study was approved by the ethical committee at Umeå University (09-175M).

### 4.2. Pre-Diagnostic Sample Cohort

Plasma samples in the pre-diagnostic cohort were obtained from the prospective NSHDS biobank [16]. The Swedish Cancer Registry was used to identify pancreatic cancer cases within the NSHDS cohort diagnosed between January 1990 and December 2009. Cases were included if EDTA plasma was available and excluded if the PDAC diagnosis was not histologically confirmed. Two controls per case were matched for sex, time of sampling (±1 year), and absence of malignant disease (Table 1).

Cases identified from NSHDS were cross-referenced with samples at the Umeå prospective clinical biobanks (UPCB) maintained by the Department of Surgery (Umeå University Hospital, Sweden) to identify those cases that had provided samples at diagnosis. Samples at diagnosis were available in the UPCB for 21 of the cases in the NSHDS cohort and these were incorporated in the cross-sectional and longitudinal analyses.

### 4.3. Samples at PDAC Diagnosis Cohort

Additional EDTA plasma samples were collected from the UPCB for a separate analysis for testing marker and model efficacy at diagnosis only. Samples collected before tumor resection from PDAC (*n* = 16), breast cancer (*n* = 9), and colorectal cancer (*n* = 10) patients were included. Samples were also included from patients with severe chronic pancreatitis (CP, *n* = 5) and age and sex-matched controls to the PDAC patients (*n* = 16) (Table 2). Samples were balanced for sex ratio (except breast cancer) and were used to construct biomarker models at the time of diagnosis.

### 4.4. ELISA and Multiplex Biomarker Assays

Plasma samples were stored at −80°C before and between enzyme-linked immunoassay (ELISA) and multiplex biomarker assay measurements. Collagen IV and endostatin were analyzed using Serum Collagen IV EIA (Argutus Medical, Dublin, Ireland) and Human Endostatin Quantikine ELISA kit (R&D Systems, Minneapolis, MN, USA), respectively. CA19-9 and CEA were measured using the multiplex bead kit WideScreen Human Cancer Panel 1 (EMD Chemicals, Gibbstown, NJ, USA). Upon manufacturer discontinuation of this kit, the equivalent Milliplex MAP Human Circulating Cancer Biomarker Magnetic Bead Panel 1 Immunoassay (EMD Millipore Corporation, Billerica, MA, USA) was substituted. Multiplex data were collected on a Bio-Plex 200 System (Bio-Rad, Hercules, CA, USA) and processed using Bio-Plex manager v4.1.1 Software (Bio-Rad). All kits were run according to the manufacturer’s protocol, using duplicate samples, and processed blinded to the study endpoint. Results with a percent coefficient of variation (CV%) less than 15 were accepted. For ELISA and multiplex assay values below the lowest reference values or normal range by kit protocol (<120 µg/L, collagen IV; 100 µg/L, endostatin; <37 U/mL, CA 19-9; <5 ng/mL, CEA), higher CV% were allowed.

### 4.5. Cross-Sectional Analysis and Model Generation

Each of the individual markers were tested using receiver operating characteristic (ROC) analysis. The combination of all four markers was modelled using orthogonal projections to latent structures discriminant analysis (OPLS-DA), following log transformation and subject to unit variance scaling. The OPLS-DA model contained one component and was tested for discrimination between case and control samples by ROC analysis. For paired or dependent analyses, the average of each pair (two controls matched to the same “case”) of controls was calculated. To compare the model with individual markers, area under the curve (AUC) values and a 95% confidence interval (95% CI) were calculated.

### 4.6. Samples at Diagnosis—Analysis and Model Generation

For samples at diagnosis unique to UPCB, individual markers were tested by ROC analysis and the combination of all four markers was modelled together in a separate OPLS-DA model following log transformation and unit variance scaling. The OPLS-DA model was tested for discrimination between PDAC and non-PDAC samples by ROC analysis. Both the model and individual markers were compared by AUC values with a 95% CI.

### 4.7. Longitudinal Analysis

Multiple pre-diagnostic NSHDS samples from the same individual (*n* = 61 cases) permitted the examination of marker expression fluctuations within individuals over time. The differences in measurement for each marker between consecutives time points was calculated, referred to as “deltas”, and plotted against time to diagnosis. In total, 108 deltas were calculated and sorted chronologically with respect to the most recent sample used for each delta calculation. Successive batches of 20 deltas were used for analysis, that is, deltas 1–20, 2–21, 3–22, … 89–108. The mean for each batch of 20 deltas was calculated and divided by the standard deviation of all case-derived deltas for each marker to generate “case delta Z-scores”. Z-scores were plotted against the time to diagnosis of the most recent sample in each batch of 20 deltas.

There were 39 individuals in the control group without PDAC that also contributed multiple NSHDS samples. These were used to control for individual fluctuations in marker expression independent of PDAC. The mean delta across all controls was subtracted from case deltas then divided by the standard deviation of all control deltas to provide a “Case vs. Control Z-score”. These values were plotted against time to diagnosis. Graphs were smoothed by a Savitzky–Golay filter.

## Figures and Tables

**Table 1:** Description of the patient and control cohort from the Northern Sweden Health and Disease Study.

Descriptor	Controls	Cases	*p* Value	Average Time from Sample Collection to Diagnosis, Years (Range)	Average Survival of Cases from Diagnosis, Days
**Number**	320	160 ^a^			
**Overall Survival**				8 (0–18)	267
**Age at initial blood sample collection**			>0.999		
<45	32	16		8 (2–18)	252
45–54	106	53		9 (0–18)	255
55–64	162	81		7 (0–18)	295
≥65	20	10		8 (1–12)	132
**Sex**			>0.999		
Male	116	58		9 (0–18)	247
Female	204	102		7 (0–18)	279
**Smoking**			0.158		
Current or previous smokers	123	72		8 (0–18)	266
Non-smokers	184	78		7 (0–17)	279
Information missing	13	10		10 (2–18)	178
**Disease stage at diagnosis**					
Confined to pancreas (I)	N/A	12		7 (2–14)	864
Locally advanced and/or regional lymph node metastasis (II)	N/A	12		7 (1–15)	413
Large vessels involved (III)	N/A	37		7 (0–17)	348
Distant metastasis (IV)	N/A	99		8 (0–18)	147
**Tumor Grade**					
Low	N/A	33		8 (0–17)	137
Intermediate	N/A	43		6 (0–15)	438
High	N/A	4		7 (2–14)	614/1314 ^b^
Information missing	N/A	80		9 (0–18)	177
**Surgical treatment**					
Curative intent	N/A	23		8 (1–15)	681
Palliative	N/A	25		7 (0–17)	159
No Surgery	N/A	111		8 (0–18)	207
Information missing	N/A	1		14 (N/A)	123
**Systemic treatment**					
Neoadjuvant and adjuvant chemotherapy	N/A	2		6 (5–7)	400
Adjuvant chemotherapy	N/A	11		11 (5–15)	312
Palliative chemotherapy	N/A	68		9 (0–18)	287
Palliative intraperitoneal 5-FU	N/A	18		8 (0–18)	339
Palliative radiotherapy	N/A	2		6 (1–10)	79
No systemic treatment	N/A	59		6 (0–17)	216
Information missing	N/A	0		N/A	N/A

^a^ Upon examination of patient charts, six were omitted from further examination because five were shown not to have PDAC, and one had another current or prior malignancy, bringing the total number of cases to 154. ^b^ The unusually high survival in this group is due to one long-term survivor. If this patient is omitted, mean survival is 614 days. *p* values calculated by chi-squared test.

**Table 2:** Description of patient samples at diagnosis from the Umeå prospective clinical biobanks.

Descriptor	PDAC (*n*)	Breast Cancer (*n*)	Colorectal Cancer (*n*)	Chronic Pancreatitis (*n*)	Controls without Malignant Disease (*n*)	*p* Value
**Patients**	16	9	10	5	16	N/A
**Age at blood sample collection**						0.117
<45	0	0	0	0	0	
45–54	3	1	0	1	3	
55–64	7	4	0	1	7	
≥65	6	4	10	3	6	
**Sex**						0.105 (0.981 ^a^)
Male	8	0	5	2	8	
Female	8	9	5	3	8	
**Disease stage at diagnosis**						0.002
i	4	6	3	N/A	N/A	
ii	0	3	3	N/A	N/A	
iii	4	0	4	N/A	N/A	
iv	8	0	0	N/A	N/A	

^a^ When sex ratio for breast cancer is excluded. *p* values calculated by chi-squared test.

## Data Availability

Data available on request due to legal restrictions. The data presented in this study are available on request from the corresponding author. The data are not publicly available due to Swedish law.
